# Serum and Cerebrospinal Fluid Cytokine Biomarkers for Diagnosis of Multiple Sclerosis

**DOI:** 10.1155/2020/2727042

**Published:** 2020-10-22

**Authors:** Ekaterina Martynova, Mehendi Goyal, Shikhar Johri, Vinay Kumar, Timur Khaibullin, Albert A. Rizvanov, Subhash Verma, Svetlana F. Khaiboullina, Manoj Baranwal

**Affiliations:** ^1^Institute of Fundamental Medicine and Biology, Kazan Federal University, Kazan, Tatarstan 420008, Russia; ^2^Department of Biotechnology, Thapar Institute of Engineering and Technology, Patiala 147004, India; ^3^Amity School of Engineering and Technology, Amity University, Jaipur, Rajasthan, India; ^4^Department of Electronics and Communication Engineering, Thapar Institute of Engineering and Technology, Patiala 147004, India; ^5^Republican Clinical Neurological Canter, Republic of Tatarstan, Russia; ^6^Department of Microbiology and Immunology, University of Nevada, Reno, NV 89557, USA

## Abstract

**Background:**

Multiple sclerosis (MS) is a chronic debilitating disorder characterized by persisting damage to the brain caused by autoreactive leukocytes. Leukocyte activation is regulated by cytokines, which are readily detected in MS serum and cerebrospinal fluid (CSF).

**Objective:**

Serum and CSF levels of forty-five cytokines were analyzed to identify MS diagnostic markers.

**Methods:**

Cytokines were analyzed using multiplex immunoassay. ANOVA-based feature and Pearson correlation coefficient scores were calculated to select the features which were used as input by machine learning models, to predict and classify MS.

**Results:**

Twenty-two and twenty cytokines were altered in CSF and serum, respectively. The MS diagnosis accuracy was ≥92% when any randomly selected five of these biomarkers were used. Interestingly, the highest accuracy (99%) of MS diagnosis was demonstrated when CCL27, IFN-*γ*, and IL-4 were part of the five selected cytokines, suggesting their important role in MS pathogenesis. Also, these binary classifier models had the accuracy in the range of 70-78% (serum) and 60-69% (CSF) to discriminate between the progressive (primary and secondary progressive) and relapsing-remitting forms of MS.

**Conclusion:**

We identified the set of cytokines from the serum and CSF that could be used for the MS diagnosis and classification.

## 1. Introduction

Multiple sclerosis (MS) is a chronic inflammatory disease of the central nervous system (CNS). It is believed that inflammation in CNS leads to myelin degradation and axon damage [1]. Studies suggest that autoreactive T lymphocytes contribute to the immune assault against myelin, neuronal death, and subsequent plaque formation [2].

Analysis of the CSF from MS patients demonstrated high levels of multiple cytokines, suggesting ongoing inflammation and leukocyte activation [3, 4]. Analysis of MS CSF revealed the activation of cytokines associated with Th1 (IFN-*γ*, TNF-*α*, and IL-2) and Th2 (IL-4, IL-5, IL-13, and IL-6) leukocytes [5]. We have shown differential activation of cytokines in CSF and serum of MS patients, where the leading role of Th1 lymphocytes in CNS MS pathogenesis was supported. Also, the upregulation of CCL27 was demonstrated in serum and CSF of MS, suggesting the role of this cytokine in neuroinflammation [6]. Although proinflammatory cytokines are commonly detected in CSF of MS, data remain limited.

The analysis of serum and CSF could identify novel biomarkers, aiding the diagnosis of various diseases and improved the therapeutic outcome. Analysis of serum and CSF samples could facilitate the early diagnosis of MS with more accuracy and cost efficiency as compared to expensive techniques such as CT or MRI scans. As an alternative approach, the machine learning methods were successfully employed for the prediction of multiple diseases, including MS [7–9]. Using this approach, we have previously identified serum cytokines which could be used for the diagnosis of MS [10].

There is no single diagnostic test for MS [11], and the current diagnosis is based on clinical symptoms and MRI data. Each of these diagnostic criteria has limitations; therefore, the identification of the novel biomarkers of the disease remains critical. Serum samples are often collected as a routine procedure for the diagnosis of MS, and they could be used to identify the disease biomarkers. We have shown multiple cytokines affected in MS [6, 10, 12]; however, these studies had limited patient group and restricted number of cytokines analyzed. In addition, we employed several machine-learning approaches to identify cytokines, which could provide high accuracy of MS diagnosis.

The present study is aimed at identifying serum and CSF cytokine-based markers for the diagnosis of MS from the panel of forty-five cytokines. ANOVA-based feature selection and Pearson correlation coefficients were done to select the most relevant cytokines from the studied panel to differentiate MS patients from non-MS. Further, five different machine learning models were developed by using selected serum and CSF cytokines to diagnose MS and identify the progressive (primary progressive MS; PPMS and secondary progressive MS: SPMS) and relapsing-remitting MS (RRMS) forms of the disease.

## 2. Materials and Methods

**Figure 1:**
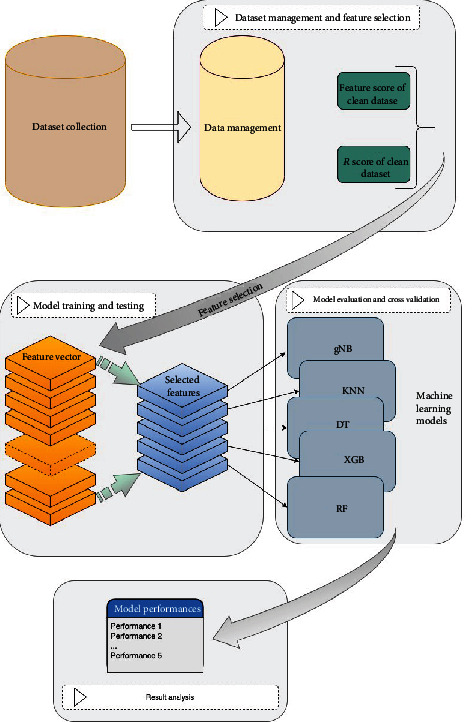
Steps of the data analysis. The study was organized into 5 stages: (1) Dataset collection. (2) Dataset managing and Feature Selection: the feature score (ANOVA) and *r* score (Pearson coefficient correlation) were calculated between: (1) serum and CSF of MS vs. non-MS; (2) serum vs. CSF of MS and non-MS. (3) Model Training and Testing: the cytokines (features) having high feature score and *r*score > 0.5 were selected for developing machine learning model. (4) Model evaluation and Cross Validation: the data of the selected features was then given as input into the five (gNB, KNN, DT, XGB, and RF) machine learning models to predict the outcome of the disease. (5) Results analysis: the performance of each model was evaluated by calculating accuracy.

The study was organized into 5 stages (Figure 1): (1) Dataset collection: the levels of different cytokines were analyzed in serum and CSF from MS patients and non-MS controls. (2) Dataset managing and Feature Selection: the feature score (ANOVA) and *r* score (Pearson coefficient correlation) were calculated between two groups (serum and CSF of MS vs. non-MS; serum vs. CSF of MS patients and non-MS controls). (3) Model Training and Testing: the cytokines (features) having high feature score and *r*score > 0.5 were selected for developing machine learning model. (4) Model evaluation and Cross Validation: the data of the selected features was then given as input into the five (gNB, KNN, DT, XGB, and RF) machine learning models to predict the outcome of the disease. (5) Results Analysis: the performance of each model was evaluated by parameters such as accuracy and AUC values Figure 1.

### 2.1. Study Subjects, Samples

One hundred one MS cases (28 males, 73 females; mean age 35.6 ± 12.52 years) admitted to the Republican Clinical Neurological Center, Republic of Tatarstan, Russian Federation, were included in this study. MS diagnosis was based on clinical presentation and brain MRI results. Serum and CSF were collected from each patient. Additionally, CSF was collected from 25 individuals, herein referred to as non-MS controls (9 males, 16 females; mean age 38.5 ± 9.2 years). Non-MS CSF samples were diagnosed with tension-type headache, residual encephalopathy, unspecified demyelinating disease of the central nervous system, cerebrovascular diseases, progressive multifocal leukoencephalopathy, and migraine with aura. Separately, serum samples were also collected from 101 non-MS controls.

### 2.2. Ethics Statement

This study was done in accordance with the recommendations of the Biomedicine Ethic Expert Committee of Republican Clinical Neurological Center, Republic of Tatarstan, Russian Federation, and the study was approved (protocol no 218, 11.15.2012) by this committee. All subjects gave written informed consent in accordance with the Declaration of Helsinki.

### 2.3. Cytokine Analysis

Serum and CSF cytokine profiles were analyzed using Bio-Plex (Bio-Rad, Hercules, CA, USA) multiplex magnetic bead-based antibody detection kits following the manufacturer's instructions. Bio-Plex Pro Human Cytokine Screening Panel, 48-Plex was used for detection for a total of 48 analytes. Serum and CSF aliquots (50 *μ*l) were used for analysis, with a minimum of 50 beads per analyte acquired. Median fluorescence intensities were measured using a Luminex 200 analyzer. Data collected was analyzed with MasterPlex CT control software and MasterPlex QT analysis software (Hitachi Software, San Bruno, CA, USA). Standard curves for each analyte were generated using standards provided by the manufacturer.

Data on forty-eight cytokine levels (IL-1*α*, IL-1*β*, IL-1Ra, IL-2, IL-2Ra, IL-3, IL-4, IL-5, IL-6, IL-7, IL-8, IL-9, IL-10, IL-12p40, IL-12(p70), IL-13, IL-16, IL-15, IL-17, IL-18, CCL2, CCL3, CCL4, CCL5, CCL7, CCL11, CCL27, CXCL1, CXCL9, CXCL10, FGF basic, G-CSF, GM-CSF, HGF, IFN*α*2, INF-*γ*, LIF, M-CSF, MIF, *β*-NGF, PDGF-bb, SDF-1a, SCF, SCGF-b, TNF-*α*, TNF-*β*, TRAIL, and VEGF) in serum (101 MS patients and 101 non-MS controls) and CSF (101 MS patients and 25 non-MS controls) was analyzed. Data on three cytokines (IL- 1Ra, IL-2ra, and IL-17) was found to be missing in some patients; therefore, these three cytokines were excluded from the dataset used in building the machine learning models.

### 2.4. Feature Selection

As the analysis of all 45 cytokines (herein referred to as features) would have increased the complexity of the machine learning algorithm, the dimensions of the dataset (feature selection) were reduced. The process of feature selection is described as the dimension reduction. The reduced dimension results in smaller dataset size and easier interpretation of data. Feature selection also benefits the reduction of the overfitting. Overfitting negatively influences the performance of the machine learning models. In order to reduce the dimensions, ANOVA-based feature selection was carried out using python software. Pearson correlation coefficient (*r*) was also calculated for each cytokine to measure the association within the two variables in python software. The *r* score represents the degree of association within the variables such that if the value lies above 0.5, then it is said to have a strong correlation. If the value lies between 0.3-0.49, it represents moderate correlation and it is said to have a low correlation if the *r* score value lies below 0.29 [13]. In the current study, we have considered the features with the *r* score values above 0.5 and significantly different at *p* < 0.05 for building the machine learning models.

### 2.5. Machine Learning Methods

Five machine-learning models, *k*-Nearest Neighbor (KNN), Decision Tree (DT), XGB (XG boost), Gaussian Naïve Bayes (gNB), and Random Forest (RF), were used. The required packages and tuning parameters to obtain the optimum results using these models are summarized in Table 1. Models were trained by considering selected cytokines to predict the target (MS vs. non-MS) and classify the progressive (primary and secondary progressive) and relapsing-remitting forms of the disease.

### 2.6. Model Evaluation

The models' performance was evaluated using accuracy [10] with the following equation:

where Accuracy = (TP + TN)/(TP + TN + FN + PP) × 100.

TN is true negative; TP is true positive; FP is false positive; FN is false negative.

AUC (Area under Curve) is area under Receiver Operating Characteristics (ROC) curve, which represents the quality of model.

### 2.7. Repeated *K*-Fold Cross Validation

*K*-fold validation [14] is used to evaluate the robustness of the proposed model. A low variation in the accuracy for successive runs of the model corresponds to robustness. The dataset was divided into equal parts to perform 15-fold cross validation; the test sets, validation sets, and training sets are chosen randomly to get a different combination of samples. The experiments are repeated three times to calculate the final accuracies.

## 3. Results

### 3.1. Clinical Presentation

MS diagnosis was established according to the 2010 Revised MacDonald's Diagnostic Criteria for MS [15]. Forty-nine cases were diagnosed with RRMS, 21 and 31 cases were diagnosed as PPMS, and SPMS, respectively (Table 2). The mean duration of the disease was 4.98 ± 6.65 years. Expanded Disability Status Scale score (EDSS) and Multiple Sclerosis Severity Score (MSSS) were 2.88 ± 1.5 and 4.5 ± 2.20, respectively. MRI detected lesions in the subcortical region, corpus callosum, and pons in MS patients. Twenty-five patients received the disease-modifying therapy while 76 patients had no treatment.

### 3.2. Analysis of Serum and CSF Cytokines in MS

**Figure 2:**
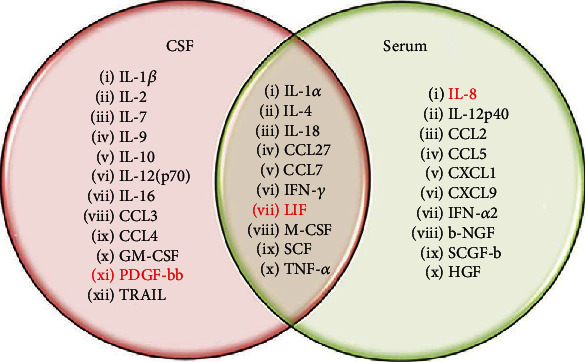
Cytokines expressed uniquely and commonly in serum and CSF of MS as compared to non-MS controls. Red highlighted cytokines are downregulated in patients as compared to control. The rest of the cytokines are upregulated in patients.

**Figure 3:**
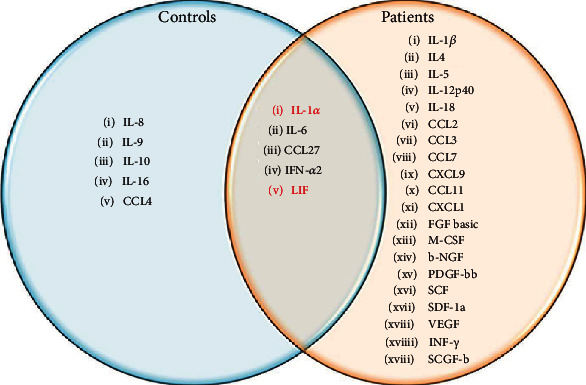
Cytokines expressed commonly and uniquely in serum and CSF of MS and non-MS controls. Red highlighted cytokines are downregulated in serum as compared to cerebrospinal fluid. The rest are upregulated in serum as compared to cerebrospinal fluid.

Levels of 45 cytokines of serum and CSF in MS and non-MS are given in Supplementary Table 1. These cytokine levels in serum and CSF from MS patients and non-MS were analyzed using the ANOVA-based feature score selection method. Subsequently, the Pearson correlation coefficient (*r* score) for each feature was calculated. Feature and *r* score data of serum and CSF cytokines in MS and non-MS are summarized in Supplementary Tables 2 and 3, respectively. Features with *r* > 0.5 were representing strong correlation between groups (serum and CSF of MS vs. non-MS; serum vs. CSF of MS and non-MS) was selected as a cutoff. Cytokines (features), having high feature score and *r* > 0.5, were used for the machine learning analysis (Figures 2 and 3). These selected cytokines were also found to be significantly different at *p* < 0.05.

Twenty-two and twenty features out of forty-five in CSF and serum, respectively, were selected based on the cut-off criteria (high feature selection score and *r* > 0.5) when MS compared to non-MS (Figure 2). Ten features (IL-1*α* IL-4, IL-18, CCL7, CCL27, CSF, IFN-*γ*, LIF, M-CSF, and TNF-*α*) were identified as commonly affected in both CSF and serum of MS, when compared to non-MS (Figure 2). Apart from the features commonly affected in MS serum and CSF, twelve features (IL-1*β*, IL-2, IL-7, IL-9, IL-10, IL- 12(p70), IL-16, CCL3, CCL4, GM-CSF, PDGF, and TRAIL) found exclusively altered in CSF in MS as compared to non-MS. Also, ten features (IL-8, IL-12p40, CCL2, CCL5, CXCL1, CXCL9, HGF, IFN-*α*2, *β*-NGF, and SCGF-b) were found uniquely altered in MS serum (Figure 2) when compared to non-MS. Additionally, the level of all selected features, except LIF (serum and CSF), PDGF-bb (CSF), IL-8 (serum), and CCL5 (serum), was found elevated in MS as compared to non-MS (Figure 2).

Differences in serum and CSF cytokine levels in MS and non-MS were also analyzed by calculating the features core and *r* score. Feature assessment revealed that levels of five cytokines (IL-1*α*, IL-6, CCL27, IFN-*α*2, and LIF) are commonly altered in serum and CSF of both MS and non-MS (Figure 3). However, more cytokines, total of twenty (IL-1*β*, IL-4, IL-5, IL12-p40, IL-18, CCL2, CCL3, CCL11, CCL27, CXCL1, CXCL9, FGF-basic, IFN-*γ*, M-CSF, *β*-NGF, PDGF-bb, SCF, SCGF-b, SDF-1a, and VEGF), were affected in serum when compared to CSF in MS. In contrast, levels of only five cytokines (IL-8, IL-9, IL-10, IL-16, and CCL4) differed in serum and CSF in non-MS (Figure 3).

**Figure 4:**
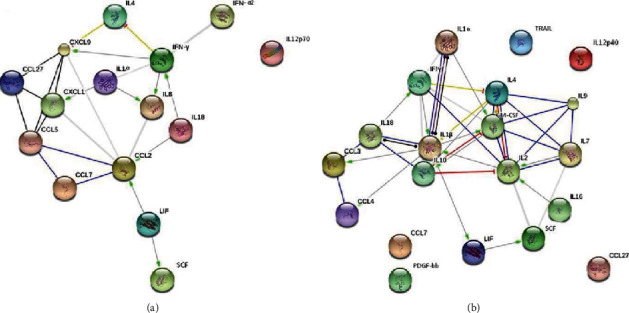
String analysis of cytokines significantly affected in serum and CSF of MS. (a) serum MS; (b) CSF MS. Green—activation; blue—binding; black—reaction; thicker the line = stronger interaction; String 9.0 (http://string91.embl.de) high confidence 0.7. (a) set of cytokines uniquely affected in serum of MS; (b) set of cytokines uniquely affected in CSF of MS.

String analysis also identified differences in the interaction of cytokines affected in serum and CSF of MS (Figures 4(a) and 4(b)).

In serum, strong interaction was found between CCL27, CCL5, CXCL1, CXCL9, CCL7, CCL2, IL-18, IL-8, HGF, and IL-4 (Figure 4(a)). A different pattern of interaction was found in cytokines affected in CSF (Figure 4(b)). In contrast to serum, where chemokine cooperation was identified, CSF samples revealed mostly interleukin (IL1*α*, IL-1*β*, IL-2, IL-4, IL-7, IL-9, IL-16, and IL-18) interaction.

### 3.3. Proposed Predictive Model

**Figure 5:**
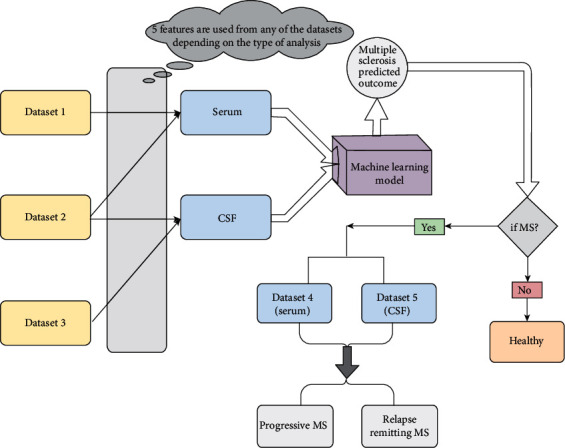
Schematic representation of the proposed model. Dataset 1: IL-8, IL-12p40, CCL2, CCL5, CXCL1, CXCL9, HGF, IFN-*α*2, b-NGF, and SCGF-b (cytokines uniquely affected in serum). Dataset 2: IL-1*α*, IL-4, IL-18, CCL7, CCL27, INF*γ*, LIF, M-CSF, TNF-*α*, and SCF (cytokines found commonly affected both in serum and CSF of MS). Dataset 3: IL-1*β*, IL-2, IL-7, IL-10, IL-9 IL-12(p70), IL-16, CCL3, CCL4, GM-CSF, PDGF-bb, and TRAIL (cytokines uniquely affected in CSF). Dataset 4 consists of dataset 1 and 2; Dataset 5 consists of dataset 2 and 3. Five cytokines from the dataset 1, 2, and 3 can be estimated in serum and CSF and then will be given as input to into the five (gNB, KNN, DT, XGB, and RF) machine learning models to predict the MS. Dataset 4 and dataset 5 in serum and CSF, respectively, can be estimated to discriminate between progressive (primary and secondary progressive) and relapse remitting forms of MS.

Five different models were trained on the dataset with the selected features to predict MS, and the proposed algorithms are depicted in Figure 5. The cytokine dataset of patients and controls was divided into training (70%) and testing (30%) dataset for both serum and CSF. Equal number of MS patients (101) and non-MS (101) controls were taken for serum analysis. However, the CSF dataset represents an unequal number of patients (101 samples) and controls (25 samples) making data unbalanced. Due to this unbalance dataset and thus to overcome the effects of overfitting, we used recursive testing.

Three independent datasets, including ten cytokines commonly affected in CSF and serum, twelve cytokines uniquely changed in CSF, and ten cytokines uniquely affected in serum (identified in Figure 3), were selected as an input to five state-of-the-art machine-learning models to predict MS.

From each cytokine datasets, five cytokines were randomly selected as an input to five machine-learning models to evaluate the accuracy in MS prediction. Interestingly, all combinations of randomly selected five cytokines have shown an accuracy of MS diagnosis ≥ 92% (Table 3).

**Figure 6:**
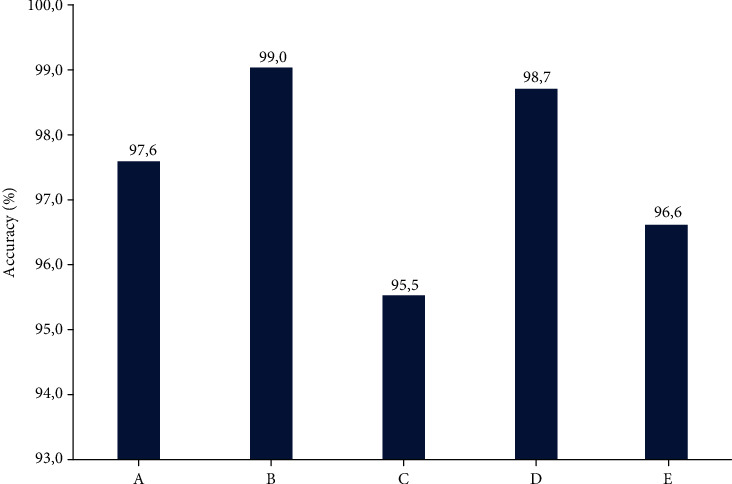
MS prediction accuracy using the KNN model with five randomly selected cytokines commonly expressed in patient serum as input. Serum common cytokines dataset consists of ten cytokines (IL-1*α*, IL-4, IL-18, CCL7, CCL27, INF*γ*, LIF, M-CSF, SCF, and TNF*α*). A: (IL-4, IL-18, CCL27, M-CSF, and SCF); B: (IL-4, CCL7, CCL27, INF*γ*, and SCF); C: (IL-1*α*, IL-18, INF*γ*, M-CSF, and TNF*α*); D: (IL-1*α*, IL-4, CCL27, INF-*γ*, and M-CSF); and E (IL-1*α*, IL-4, IL-18, LIF, and M-CSF) are datasets which are generated by considering five random cytokines. The accuracy is the average of 15 runs.

An example of the accuracy of MS diagnosis is presented by using KNN, one of the models tested, for randomly selected five serum cytokines differentially expressed in MS serum (Figure 6).

**Figure 7:**
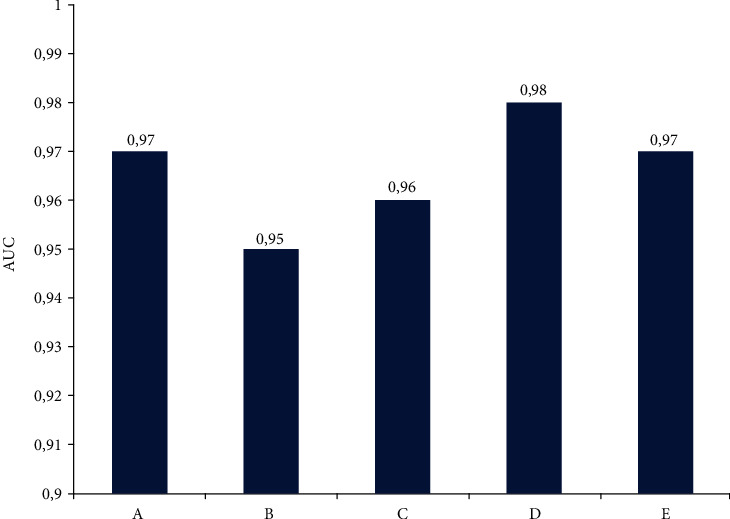
AUC based on five random cytokines commonly expressed in serum generated by gNB models to diagnose MS. Serum common cytokines dataset consists of ten cytokines (IL-1*α*, IL-4, IL-18, INF*γ*, CCL7, CCL27, LIF, M- CSF, SCF, and TNF*α*). A: (IL-18, CCL27, INF-, LIF, and TNF*α*); B: (IL-4, CCL7, CCL27, SCF, and TNF*α*); C: (IL-4, IL-18, CCL27, SCF, and TNF*α*); D: (CCL7, CCL27, INF-*γ*, M-CSF, and TNF-*α*); and E (IL-4, IL-18, INF-*γ*, LIF, and SCF) are datasets which are generated by considering five random cytokines. AUC is the average of 15 runs.

Interestingly, the accuracy of MS diagnosis was the highest, reaching 99%, when cytokines CCL27, IL-4 and IFN-*γ* were included into the randomly selected five cytokines. Area under the Curve (AUC), which represents the reliability of the model, is also demonstrated using gNB, one of the randomly generated model using five serum cytokines. This AUC value was more than 0.95, indicating high reliability of the model (Figure 7).

All five machine learning models (KNN, DT, XGB, gNB, and RF) demonstrated relatively similar accuracy indicating that any of them can be used for the prediction of MS (Table 3).

Twenty and twenty-two cytokines affected in serum and CSF, respectively, were also taken as input to classify progressive (PPMS and SPMS) and RRMS forms of the disease. The accuracy was found to be in the range of 70-78% and 60-69% for serum and CSF, respectively (Table 4).

## 4. Discussion

Diagnosis of MS remains a challenge, as the disease has multiple clinical forms and symptoms could relapse and disappear [16]. Changes in a large number of cytokines, a soluble biomarkers of inflammation and leukocyte activation, in serum and CSF were demonstrated [6, 12]. These data suggest that some cytokines could have a diagnostic and prognostic value in MS [10]. We have previously applied the machine learning models to diagnose MS, using limited data on cytokines affected in the serum of MS [10], which produced relatively low confidence result. Therefore, we expanded the number of cytokines (total of 45), which included interleukins, growth factors, and chemokines, so the computational data analysis would identify the group of biomarkers differentiating MS with a high level of confidence.

We found that twenty-two and twenty cytokines in CSF and serum, respectively, were affected (strong correlation, *r* > 0.5) in MS as compared to non-MS. All five models have shown the high accuracy of MS diagnosis, ranging between 90-99%. In our previous report [10], four models (SVM, DT, RF, and KNN) were applied and only RF model has shown the accuracy of 90.91% with limited cytokine dataset (eight cytokines). In the present analysis, the RF model has shown the accuracy of ≥92%, which is similar to our previous report [10]. As compared to the basic models (KNN, DT, gNB, and RF), XGB uses the ensemble approach [17]. XG also demonstrated remarkable accuracy, ranging between 92 and 98%. Seven (IL-1*β*, IL-2, IL-4, IL-8, IL-10, IFN-*γ*, and TNF-*α*) out of eight cytokines used in the previous report [10] were also found in the current study, indicating the reliability of the published and current results. However, by using the larger number of biomarkers, we were able to determine that the minimum of five cytokines is required to achieve the highest accuracy of MS diagnosis. Also, these models were able to identify PPMS, SPMS, and RRMS forms of the disease by taking input of twenty (serum) and twenty-two (CSF) cytokines with an accuracy of 70-78% and 60-69%, respectively.

When five cytokines were randomly selected, the group including CCL27, IL-4, and IFN-*γ* combined with any other three cytokines demonstrated the highest precision of MS differentiation from non-MS. This data corroborated our previous report on the potential role of CCL27 in MS pathogenesis [18]. Originally identified in skin [19], the expression of this cytokine was demonstrated in brain neuroglia [20]. It was shown that CCL27 could enhance the inflammation by releasing IL-4 [21]. Studies also have demonstrated that CCL27 could trigger T memory cells [22] to produce IL-4 and IFN-*γ* [23]. Although these data provide limited evidence on the link between CCL27 and MS pathology, our observation of the high level of this cytokine in MS serum and CSF suggests its role in the pathogenesis of the disease. This assumption was confirmed by Monfared et al., demonstrating the increased serum CCL27 level in MS [24].

One of the interesting observations was that some cytokines are differentially affected in serum and CSF in MS. IL-1*α*, IL-1*β*, and IL-18, affected in MS CSF, were linked to strong inflammatory reaction [25]. Two of these cytokines, IL-1*β* and IL-18, are the product of activated inflammasome, regulating inflammatory response [26]. These findings support the key role of inflammation in brain pathology and, also, supports the use of inflammasome inhibitors as therapeutic for MS [27]. In addition to inflammation, interleukins and chemokines affected in serum and CSF could direct leukocyte migration targeting Th1 cells [28].

## 5. Conclusion

We have identified ten cytokines (IL-1*α*, IL-4, IL-18, CCL7 CCL27, INF-*γ*, LIF, M- CSF, SCF, and TNF-*α*) which can be analyzed either in serum or CSF to differentiate MS from non-MS. Also, the random selection of any five cytokines from the dataset of altered cytokines in serum and CSF could diagnose MS with an accuracy between 90 and 99%. Interestingly, the highest accuracy of MS diagnosis (99%) was demonstrated when CCL27, IL-4, and IFN-*γ* were selected, suggesting their important role in MS pathogenesis. Also, the accuracy of models to identify progressive (PPMS and SPMS) and RRMS was 70-78% (serum) and 60-69% (CSF).

## Figures and Tables

**Table 1:** Tuning parameters of the machine learning models.

Model	Method	Tuning parameter
Gaussian Naïve Bayes (gNB)	GaussianNB	var_smoothing = 1*e* − 09
*k*-Nearest Neighbor (KNN)	KNN_model	leaf_size = 30, metric = ^“^minkowski^”^, n_neighbors = 5, *p* = 2
Decision tree (DT),	DecisionTreeClassifier	Criterion = ^“^gini^”^, max_depth = 2, presort = ^“^deprecated^”^, splitter = ^“^best^”^
XGB (XG boost)	XGBClassifier	Booster = ^“^gbtree^”^, learning_rate = 0.1, max_depth = 3, n_estimators = 200, objective = ^“^binary : logistic^”^, verbosity = 1
Random forest (RF) (RandomForestClassifier)	RandomForestClassifier	Criterion = ^“^gini^”^, n_estimators = 50

**Table 2:** Demographic and clinical details of MS patients.

Characteristics		Number or mean ± SD
Age		35.61 ± 12.52
Gender	Male	28
	Female	73
MS type	Relapse remitting	49
	Primary progressive	21
	Secondary progressive	31
Disease duration		4.98 ± 6.65
EDSS		2.88 ± 1.53
MSSS		4.5 ± 2.197
Patients on treatment		25
Patient without treatment		76

**Table 3:** Different model accuracy of MS diagnosis.

Cytokines datasets		Model (accuracy in percentage)				
		gNB	KNN	DT	XGB	RF
IL-1*α*, IL-4, IL-18, CCL7, CCL27, INF*γ*, LIF, M- CSF, TNF-*α*, SCF^∗^	CSF	≥96	≥93	≥93	≥92	≥92
	Serum	≥95	≥95	≥96	≥96	≥96
IL-1*β*, IL-2, IL-7, IL-10, IL-9 IL-12(p70), IL-16, CCL3, CCL4, GM-CSF, PDGF-bb, TRAIL^∗∗^	CSF	≥96	99	≥96	≥98	99
IL-8, IL-12p40, CCL2, CCL5, CXCL1, CXCL9, HGF, IFN-*α*2, b-NGF, SCGF-b^∗∗∗^	Serum	≥92	≥90	≥92	≥94	≥95

In each cytokine dataset, randomly five cytokines were taken in each model to calculate the accuracy with *k*-fold cross validation. ^∗^Cytokines found commonly affected in serum and CSF of MS; ^∗∗^Cytokines uniquely affected in CSF; ^∗∗∗^Cytokines uniquely affected in serum.

**Table 4:** Different model accuracy to classify MS.

Cytokines datasets		Model (accuracy in percentage)				
		gNB	KNN	DT	XGB	RF
IL-1*α*, IL-4, IL-8, IL-12p40, IL-18, CCL2, CCL5, CCL7, CCL27, CXCL1, CXCL9, HGF, INF-*γ*, IFN-*α*2, TNF-*α*, LIF, b-NGF, SCGF-b M-CSF, SCF^∗^	Serum	70	72	74	78	73
IL-1*α*, IL-1*β*, IL-2, IL-4, IL-7, IL-9, IL-10, IL- 12(p70), IL-16, IL-18, CCL3, CCL4, CCL7, CCL27, GM-CSF, INF-*γ*, TNF-*α*, LIF, M-CSF, SCF, PDGF- bb, TRAIL^∗∗^	CSF	69	65	65	60	63

^∗^ Cytokines found altered serum of MS. ^∗∗^Cytokines found altered in CSF of MS.

## Data Availability

The corresponding author has full access to data and will share it with requesting researchers on request after signing an agreement stating not to use data for any purpose other than intended research.

## Supplementary Materials

Supplementary Materials Supplementary Table 1: cytokine level of cerebral spinal fluid and serum in MS patients and control. Supplementary Table 2: feature score and Pearson correlation coefficient (*r*) values for serum and CSF cytokines in MS as compared to non-MS control. Supplementary Table 3: feature score and Pearson correlation coefficient (*r*) values for serum as compared to CSF cytokines in MS and non-MS control.
