# Supplementary material for: Serum and Cerebrospinal Fluid Cytokine Biomarkers for Diagnosis of Multiple Sclerosis
Source: Mediators Inflamm. 2020 Oct 22;2020:2727042. doi: 10.1155/2020/2727042 (PMC7607285; doi:10.1155/2020/2727042)
Supplement: Supplementary Materials — Supplementary Table 1: cytokine level of cerebral spinal fluid and serum in MS patients and control. Supplementary Table 2: feature score and Pearson correlation coefficient (r) values for serum and CSF cytokines in MS as compared to non-MS control. Supplementary Table 3: feature score and Pearson correlation coefficient (r) values for serum as compared to CSF cytokines in MS and non-MS control. [file 2727042.f1.docx]

Supplementary Table 1: Cytokines level of cerebral spinal fluid and serum in MS patients and control

| Cytokine | Cerebral spinal fluid (pg/ml) | | Serum (pg/ml) | |
| --- | --- | --- | --- | --- |
|  | MS-Patient | Control | MS-Patient | Control |
| CXCL9 | 85.35±61.17* | 53.96± 230.95 | 947.99±469.18 | 283.45±535.95 |
| CCL4 | 91.79±53.62 | 5.67±2.337 | 62.07±30.55 | 114.32±79.86 |
| IL-6 | 3.05±1.65 | 2.14±1.73 | 68.38±49.01 | 66.26±28.19 |
| IFN-α2 | 4.2±1.46 | 2.91±1.18 | 37.85±7.59 | 25.22±8.57 |
| IFN-γ | 511.89±211.21 | 4.29±1.33 | 138.69±103.44 | 17.25±22.84 |
| SDF- 1a | 75.39±35.63 | 57.57±17.58 | 2870.11±1342.89 | 1865.19±1752.96 |
| CCL7 | 12.28±2.76 | 7.91±2.65 | 379.51±229.85 | 50.92±38.49 |
| IL-16 | 93.79±38.79 | 8.74±7.36 | 107.96±88.68 | 135.67±89.38 |
| IL12p40 | 47.66±22.35 | 57.75±10.28 | 3610.79±2577.19 | 36.27±34.96 |
| LIF | 8.99±4.08 | 18.52±3.83 | 23.19±8.11 | 3.21±3.28 |
| TNF-β | 4.15±1.45 | 5.43±2.43 | 8.59±6.26 | 3.62±2.80 |
| IL-5 | 18.8±4.98 | 18.88±3.18 | 88.84±61.23 | 83.92±109.05 |
| GM-CSF | 157.47±85.19 | 2.83±0.84 | 80.38±50.02 | 77.28±128.59 |
| MIF | 384.92±226.96 | 166.68±208.57 | 306.07±287.51 | 533.88±1034.12 |
| TNF-α | 35.41±10.59 | 11.15±4.02 | 71.63±53.77 | 8.25±12.99 |
| CCL5 | 220.71±178.60 | 84.72±60.68 | 414.31±288.91 | 1424.27±1055.54 |
| IL-2 | 44.84±14.56 | 7.67±1.75 | 79.48±52.98 | 64.44±63.95 |
| IL1β | 29.05±11.04 | 1.04±0.53 | 12.08±6.58 | 8.92±7.08 |
| IL-18 | 18.89±9.45 | 1.78±0.55 | 46.89±17.45 | 11.80±16.69 |
| CCL11 | 78.38±69.26 | 5.45±5.69 | 281.89±186.89 | 399.5±376.44 |
| FGF basic | 29.09±14.67 | 15.09±2.28 | 95.99±57.68 | 91.55±129.69 |
| VEGF | 47.37±33.18 | 27.50±6.81 | 183.17±65.97 | 184.82±164.00 |
| b-NGF | 2.55±13.21 | 1.66±0.46 | 352.34±187.58 | 4.63±5.78 |
| PDGF-bb | 16.09±55.08 | 170.43±134.13 | 1258.30±1027.94 | 1025.83±684.55 |
| CXCL10 | 1476.06±1462.58 | 347.06±263.36 | 295.97±139.83 | 333.68±345.36 |
| IL-13 | 31.01±23.89 | 1.82±0.73 | 74.08±60.32 | 45.74±82.18 |
| IL-4 | 15.67±4.75 | 4.27±1.38 | 41.54±16.09 | 15.77±9.86 |
| CCL2 | 196.82±117.23 | 120.67±65.41 | 526.76±230.88 | 202.76±140.33 |
| IL-8 | 117.33±96.27 | 3.77±1.14 | 156.06±122.11 | 587.00±321.05 |
| CCL3 | 23.24±11.93 | 0.333±0.18 | 80.95±60.69 | 109.05±85.48 |
| IL-10 | 15.4±4.39 | 3.65±1.12 | 25.41±20.87 | 51.62±36.09 |
| G-CSF | 32.11±14.52 | 28.37±5.85 | 68.79±138.75 | 124.34±128.32 |
| CXCL1 | 57.41±23.92 | 60.11±13.65 | 151.31±95.96 | 41.26±41.19 |
| HGF | 200.39±137.14 | 134.02±83.65 | 496.91±341.82 | 90.97±114.88 |
| IL-1α | 216.18±74.79 | 16.73±3.87 | 4.78±2.76 | 1.33±1.52 |
| IL-3 | 10.43±6.07 | 3.46±2.38 | 18.07±13.65 | 24.15±36.21 |
| SCF | 13.06±4.36 | 5.98±1.91 | 167.34±113.95 | 33.54±38.22 |
| TRAIL | 11.29±3.32 | 3.20±0.98 | 170.12±231.22 | 13.42±14.05 |
| M-CSF | 18.94±4.32 | 7.44±6.39 | 279.06±2220.22 | 6.34±9.81 |
| CCL27 | 29.84±8.93 | 5.12±2.59 | 2877.43±1693.58 | 294.64±204.90 |
| IL-7 | 33.13±8.55 | 8.99±3.93 | 53.12±35.82 | 62.45±87.59 |
| IL-12p70 | 92.84±50.73 | 7.56±2.21 | 158.10±102.68 | 208.72±209.73 |
| IL-9 | 106.81±65.46 | 1.64±0.75 | 138.66±90.14 | 136.86±93.01 |
| SCGF-b | 3284.662±2195.89 | 4323.66±3027.69 | 17472.21±14805.04 | 4250.83±3685.79 |
| IL-15 | 150.39±51 | 107.72±32.87 | 95.47±69.39 | 101.01±102.23 |

* Data is given as mean±SD

Supplementary Table 2: Feature score and Pearson correlation coefficient(r) values for serum and CSF cytokines in MS as compared to non-MS control

| Cerebral spinal fluid | | |  | Serum | |
| --- | --- | --- | --- | --- | --- |
| Features | R values | Feature score | Features | R-Value | Feature score |
| CCL27 | 0.776 | 187.7269 | LIF | 0.8515 | 527.6176 |
| IL-7 | 0.7706 | 181.2567 | β-NGF | 0.7964 | 346.7087 |
| IL-1α | 0.7666 | 176.7852 | CCL27 | 0.7325 | 231.5134 |
| IL-10 | 0.7638 | 173.6329 | IL-18 | 0.7183 | 213.202 |
| IL-2 | 0.7515 | 160.9191 | CCL7 | 0.7078 | 200.7716 |
| IL-1β | 0.7502 | 159.6129 | IL-12p40 | 0.7019 | 194.2616 |
| TRAIL | 0.7365 | 147.0136 | IL-4 | 0.6962 | 188.0834 |
| INF-γ | 0.7325 | 143.5489 | IL-8 | 0.6655 | 158.9732 |
| IL-4 | 0.7289 | 140.5204 | M-CSF | 0.6603 | 154.5892 |
| PDGF-bb | 0.7256 | 137.8682 | CCL2 | 0.6486 | 145.2425 |
| TNF-α | 0.7111 | 126.8574 | INF-γ | 0.6317 | 132.7803 |
| M-CSF | 0.7011 | 119.8437 | TNF-α | 0.6314 | 132.5614 |
| IL-16 | 0.6995 | 118.7888 | HGF | 0.6247 | 127.9911 |
| LIF | 0.6897 | 112.4737 | SCF | 0.6205 | 125.1836 |
| CCL3 | 0.652 | 91.69869 | IFN- α2 | 0.6168 | 122.7795 |
| GM-CSF | 0.6306 | 81.86804 | IL-1α | 0.6137 | 120.8455 |
| IL-18 | 0.6299 | 81.55548 | CXCL1 | 0.5984 | 111.5534 |
| IL-12(p70) | 0.6013 | 70.2422 | CXCL9 | 0.5526 | 87.91106 |
| CCL4 | 0.5842 | 64.2352 | CCL5 | 0.5484 | 86.01974 |
| IL-9 | 0.5841 | 64.21113 | SCGF-b | 0.5244 | 75.84721 |
| SCF | 0.5831 | 63.88159 | TNF-β | 0.4555 | 52.34517 |
| CCL7 | 0.544 | 52.11152 | TRAIL | 0.4333 | 46.2231 |
| IL-13 | 0.4799 | 37.10023 | IL-10 | 0.4079 | 39.91701 |
| IL-8 | 0.4669 | 34.56953 | CCL4 | 0.3984 | 37.72278 |
| IL-3 | 0.4517 | 31.77675 | SDF-1a | 0.3077 | 20.9168 |
| CCL11 | 0.4266 | 27.58358 | IL-1β | 0.2266 | 10.8283 |
| FGF basic | 0.3928 | 22.61603 | G-CSF | 0.2045 | 8.724916 |
| MIF | 0.3725 | 19.9816 | CCL11 | 0.1951 | 7.917662 |
| IFN-α2 | 0.3499 | 17.29971 | IL-13 | 0.1938 | 7.806697 |
| IL-15 | 0.3234 | 14.4849 | CCL3 | 0.1871 | 7.254759 |
| CXCL10 | 0.323 | 14.4446 | IL-16 | 0.1545 | 4.88964 |
| CCL5 | 0.319 | 14.04362 | IL-12(p70) | 0.1523 | 4.746692 |
| TNF-β | 0.2963 | 11.93681 | MIF | 0.1491 | 4.549861 |
| CCL2 | 0.2702 | 9.763754 | PDGF-bb | 0.1326 | 3.578866 |
| VEGF | 0.2602 | 9.003136 | IL-2 | 0.1277 | 3.313042 |
| IL-6 | 0.223 | 6.486295 | IL-3 | 0.111 | 2.494825 |
| SDF-1a | 0.2191 | 6.255608 | CXCL10 | 0.0717 | 1.034857 |
| HGF | 0.2098 | 5.708127 | IL-7 | 0.0699 | 0.981961 |
| IL-12p40 | 0.1905 | 4.669759 | IL-15 | 0.0318 | 0.202785 |
| SCGF-b | 0.1513 | 2.906303 | IL-5 | 0.028 | 0.156489 |
| G-CSF | 0.1205 | 1.826198 | IL-6 | 0.0266 | 0.14115 |
| CXCL9 | 0.117 | 1.721046 | FGF basic | 0.0223 | 0.099149 |
| CXCL1 | 0.0474 | 0.279579 | GM-CSF | 0.016 | 0.051042 |
| b-NGF | 0.0303 | 0.113907 | IL-9 | 0.0099 | 0.019577 |
| IL-5 | 0.0049 | 0.002966 | VEGF | 0.0067 | 0.008847 |

Supplementary table 3: Feature score and Pearson correlation coefficient(r) values for serum as compared to CSF cytokines in MS and non-MS control.

| Serum-cerebral spinal fluid Controls | | | Serum-cerebral spinal fluid MS patients | | |
| --- | --- | --- | --- | --- | --- |
| Features | R values | Feature score | Features | R-Value | Feature score |
| IL-1α | 0.9432 | 999.6047 | IFN-α2 | 0.9514 | 1910.793 |
| LIF | 0.8767 | 412.0623 | IL-1α | 0.8951 | 805.9161 |
| IFN-α2 | 0.7585 | 167.9707 | SDF-1a | 0.8283 | 437.1264 |
| IL-6 | 0.7134 | 128.5254 | β-NGF | 0.7975 | 349.4628 |
| IL-8 | 0.6309 | 82.00268 | VEGF | 0.7942 | 341.5734 |
| IL-9 | 0.5456 | 52.55742 | CXCL9 | 0.7916 | 335.7345 |
| IL-16 | 0.5365 | 50.1287 | CCL27 | 0.7669 | 285.5304 |
| CCL27 | 0.5346 | 49.63049 | CCL7 | 0.7504 | 257.763 |
| CCL4 | 0.5205 | 46.0736 | INF-γ | 0.7482 | 254.3385 |
| IL-10 | 0.511 | 43.82514 | LIF | 0.7435 | 247.2862 |
| CCL3 | 0.4948 | 40.1994 | IL-4 | 0.7384 | 239.7306 |
| CCL5 | 0.4938 | 39.99255 | IL-18 | 0.708 | 200.9891 |
| PDGF-bb | 0.4874 | 38.63289 | IL-12p40 | 0.7008 | 193.0468 |
| IL-4 | 0.4616 | 33.57243 | SCF | 0.6931 | 184.8994 |
| CCL7 | 0.4475 | 31.04121 | IL-6 | 0.6875 | 179.2295 |
| IL-1β | 0.4441 | 30.4602 | IL-1β | 0.6843 | 176.1687 |
| CCL11 | 0.4245 | 27.25046 | CCL2 | 0.6712 | 163.973 |
| SDF-1a | 0.4192 | 26.43858 | PDGF-bb | 0.6515 | 147.4931 |
| VEGF | 0.3949 | 22.91409 | M-CSF | 0.6429 | 140.8688 |
| IL12p70 | 0.3946 | 22.86574 | IL-5 | 0.6295 | 131.296 |
| IL-2 | 0.3689 | 19.53757 | FGF basic | 0.6241 | 127.6183 |
| G-CSF | 0.3182 | 13.96905 | CCL11 | 0.5873 | 105.2981 |
| TRAIL | 0.3115 | 13.32195 | CXCL1 | 0.5593 | 91.03604 |
| SCF | 0.3078 | 12.97596 | SCGF-b | 0.5587 | 90.75415 |
| IL-7 | 0.2618 | 9.125928 | CCL3 | 0.5526 | 87.93181 |
| IL-12p40 | 0.2609 | 9.053331 | HGF | 0.4966 | 65.46595 |
| IL-18 | 0.2598 | 8.977938 | CXCL10 | 0.4957 | 65.15702 |
| TNF-β | 0.2587 | 8.894535 | GM-CSF | 0.485 | 61.50532 |
| IL-5 | 0.258 | 8.843128 | TRAIL | 0.4386 | 47.64866 |
| FGF basic | 0.2553 | 8.644464 | TNF-β | 0.4385 | 47.60011 |
| GM-CSF | 0.2509 | 8.32722 | IL-13 | 0.4266 | 44.50162 |
| IL-3 | 0.2478 | 8.114814 | TNF-α | 0.4251 | 44.11291 |
| CCL2 | 0.2474 | 8.081118 | IL-2 | 0.4089 | 40.14544 |
| IFN-γ | 0.2466 | 8.030783 | IL-15 | 0.4069 | 39.67740 |
| IL-13 | 0.2327 | 7.100776 | CCL5 | 0.3754 | 32.81426 |
| β-NGF | 0.224 | 6.552675 | IL-12(p70) | 0.3753 | 32.78993 |
| CXCL1 | 0.1946 | 4.881657 | IL-7 | 0.36 | 29.77027 |
| CXCL9 | 0.186 | 4.443074 | IL-3 | 0.3413 | 26.36681 |
| MIF | 0.1581 | 3.17812 | CCL4 | 0.3239 | 23.43382 |
| HGF | 0.1486 | 2.800672 | IL-10 | 0.3164 | 22.2435 |
| TNF-α | 0.0958 | 1.149382 | IL-9 | 0.1991 | 8.253328 |
| M-CSF | 0.0398 | 0.196471 | G-CSF | 0.1837 | 6.981336 |
| IL-15 | 0.029 | 0.10469 | IL-8 | 0.1743 | 6.26693 |
| CXCL10 | 0.0279 | 0.096612 | MIF | 0.1512 | 4.681141 |
| SCGF-b | 0.0067 | 0.005496 | IL-16 | 0.1035 | 2.163769 |
